# Adult mouse eIF2Bε Arg191His astrocytes display a normal integrated stress response *in vitro*

**DOI:** 10.1038/s41598-018-21885-x

**Published:** 2018-02-28

**Authors:** Lisanne E. Wisse, Timo J. ter Braak, Malu-Clair van de Beek, Carola G. M. van Berkel, Joke Wortel, Vivi M. Heine, Chris G. Proud, Marjo S. van der Knaap, Truus E. M. Abbink

**Affiliations:** 1 Department of Pediatrics/Child Neurology, VU University Medical Center, Amsterdam, The Netherlands; 2 Department of Functional Genomics, VU University Amsterdam, Amsterdam, The Netherlands; 3 Department of Complex Trait Genetics, VU University Amsterdam, Amsterdam, The Netherlands; 4 Centre for Biological Sciences, University of Southampton, Southampton, United Kingdom; 5 Present Address: Laboratory Genetic Metabolic Diseases, Departments of Pediatrics and Clinical Chemistry, Amsterdam Medical Center, Amsterdam, The Netherlands; 6 Present Address: South Australian Health and Medical Research Institute, University of Adelaide, Adelaide, Australia

## Abstract

Vanishing white matter (VWM) is a genetic childhood white matter disorder, characterized by chronic as well as episodic, stress provoked, neurological deterioration. Treatment is unavailable and patients often die within a few years after onset. VWM is caused by recessive mutations in the eukaryotic initiation factor 2B (eIF2B). eIF2B regulates protein synthesis rates in every cell of the body. In normal cells, various types of cellular stress inhibit eIF2B activity and induce the integrated stress response (ISR). We have developed a VWM mouse model homozygous for the pathogenic Arg191His mutation in eIF2Bε (*2b5*^*ho*^), representative of the human disease. Neuropathological examination of VWM patient and mouse brain tissue suggests that astrocytes are primarily affected. We hypothesized that VWM astrocytes are selectively hypersensitive to ISR induction, resulting in a heightened response. We cultured astrocytes from wildtype and VWM mice and investigated the ISR in assays that measure transcriptional induction of stress genes, protein synthesis rates and cell viability. We investigated the effects of short- and long-term stress as well as stress recovery. We detected congruent results amongst the various assays and did not detect a hyperactive ISR in VWM mouse astrocytes.

## Introduction

Vanishing white matter (VWM) is one of the more prevalent inherited childhood brain white matter disorders^1^. Patients show signs of chronic as well as episodic neurological deterioration^2^. Episodes of fast deterioration are provoked by stresses, such as minor head trauma and febrile infections^2,3^. The onset of the disease varies, but is mostly observed in children with an age below 6 years^4^. Postmortem neuropathological examination shows selective involvement of the brain white matter, whereas grey matter structures are spared. Within the white matter, astrocytes and oligodendrocytes (together the “macroglia” or, in short, the “glia”) are selectively affected. They have abnormal morphology^5,6^, are immature^5–8^ and fail in their mature functions, leading to a profound lack of myelin (oligodendrocyte function) and to deficient scar tissue formation (astrocyte function)^5,9^.

VWM patients have bi-allelic recessive mutations in any of the five genes encoding the subunits of the eukaryotic translation factor 2B (eIF2B)^10,11^. We recently developed a VWM mouse model with a homozygous Arg191His mutation in eIF2Bε (*2b5*^*ho*^), representing the human Arg195His mutation^12^. This founder mutation in the Cree population in North America^13^ causes a severe variant of VWM^14^. The “*2b5*^*ho*^ mouse model” recapitulates the human disease, with neurological dysfunction as well as astrocyte and oligodendrocyte abnormalities, similar to those observed in patients^12^. Astrocyte dysfunction is most likely the primary one with oligodendrocyte dysfunction being secondary^12^.

eIF2B functions as guanine nucleotide exchange factor (GEF), exchanging GDP for GTP on its trimeric substrate eIF2^15^. eIF2-GTP binds a charged initiator methionyl-transfer RNA constituting the ternary complex. This complex binds to the small ribosomal subunit, which binds to and scans the 5′untranslated region. Upon start codon recognition and delivery of the methioninyl-tRNA, eIF2-GTP is hydrolyzed to inactive eIF2-GDP, which leaves the ribosome. eIF2B reactivates eIF2-GDP to eIF2-GTP; a new ternary complex can form and initiate a next round of translation. In this whole process, eIF2B activity is a key regulator, determining the translation rate, especially under proteotoxic stress conditions^15^. Regulation of eIF2B activity occurs in several ways. Most studied is the regulation by phosphorylation of serine 51 (Ser51) of the eIF2α subunit, which leads to activation of the integrated stress response (ISR). Four kinases are known that phosphorylate Ser51 upon specific triggers associated with proteotoxicity^16^: protein kinase activated by double-stranded RNA (PKR), PKR-like endoplasmic reticulum kinase (PERK), general control non-derepressible 2 (GCN2) and heme-regulated inhibitor (HRI). PERK is activated by unfolded proteins^17^. PKR is activated by accumulation of double-stranded RNA during infection. GCN2 is activated by uncharged tRNAs when amino acids are low^17^. HRI is activated during heme deprivation and oxidative stress^17^. eIF2α phosphorylated at Ser51 binds tightly to eIF2B and acts as a competitive inhibitor^15^. Consequently, general protein synthesis is decreased. Translation of specific proteins is increased upon eIF2α phosphorylation, with activating transcription factor 4 (ATF4) as a prototypic example^18,19^. ATF4 induces the transcription of genes encoding proteins such as C/EBP homologous protein (CHOP)^20^, growth arrest and DNA damage-inducible protein 34 (GADD34)^21^ and Tribbles homolog 3 (TRIB3)^20,22^.

Previously we reported that VWM patients’ brains show activation of the unfolded protein response (UPR) in glia^23,24^. The UPR is activated upon endoplasmic reticulum (ER) stress, which activates three independent sensors in the ER membrane: PERK, activating transcription factor 6 (ATF6) and Inositol-requiring enzyme 1α (IRE1α). Upon ER stress, PERK dimerizes, autophosphorylates and promotes the ISR^20^. In addition, ATF6 leaves the ER and migrates towards the Golgi where it is processed into ATF6-c, an active transcription factor that induces expression of the gene encoding protein disulfide isomerase family A member 4 (*Pdia4*) and others^25^. Furthermore, IRE1α dimerizes, autophosphorylates and induces the synthesis of the transcription factor X-box-binding protein 1 s (XBP1s) through a cytoplasmic splicing event of the unspliced mRNA precursor *XBP1u*^26^. Each transcription factor selectively induces transcription of effector genes that allow cells to cope with the stressor, helping to restore ER function to normal or induce apoptosis if the stressor is too severe^27^.

We and others have previously hypothesized that eIF2B mutations lead to hypersensitivity of the ISR and have investigated this hypothesis in patients’ lymphoblasts and fibroblasts with thapsigargin or heat shock as stress inducers for the ISR^28–30^. Others have addressed the hypothesis by studying UPR or ISR induction in rat and human oligodendrocyte cell lines or hamster ovary cell lines^31–34^. We and others have shown normal stress responses in cells with mutated eIF2B^28,29,31^. In contrast, one study showed an increased ISR in response to thapsigargin in VWM patients’ fibroblasts, which was abrogated upon viral transformation^30^. Two further studies showed increased ISR and decreased cell viability in oligodendrocyte cell lines in response to thapsigargin^32,34^. All in all, the hypothesized hypersensitivity to ISR activation was not substantiated in all cultured cells with eIF2B mutations. Importantly, none of the studies addressed the stress response in astrocytes, which are likely primarily affected in VWM. In this study, we investigated if the stress response in primary *2b5*^*ho*^ mouse astrocytes is hypersensitive resulting in a more pronounced increase in ATF4-induced mRNA markers, reduction of the protein synthesis rates and cell viability.

The *2b5*^*ho*^ mouse model allows investigation of the UPR and ISR in primary astrocytes obtained from wild type (wt) and *2b5*^*ho*^ mouse brains^12,35^. We selectively induced the UPR or ISR with specific compounds. We assessed the astrocytic UPR at molecular and cellular levels following short-term (4 hours (h)) and long-term (16 h – 96 h) treatments. Recovery from UPR was also studied. We found that *2b5*^*ho*^ astrocytes responded similarly as wt astrocytes in all assays.

## Results

### Wt and *2b5*^*ho*^ astrocytes respond similarly to short term UPR or ISR induction

**Figure 1:**
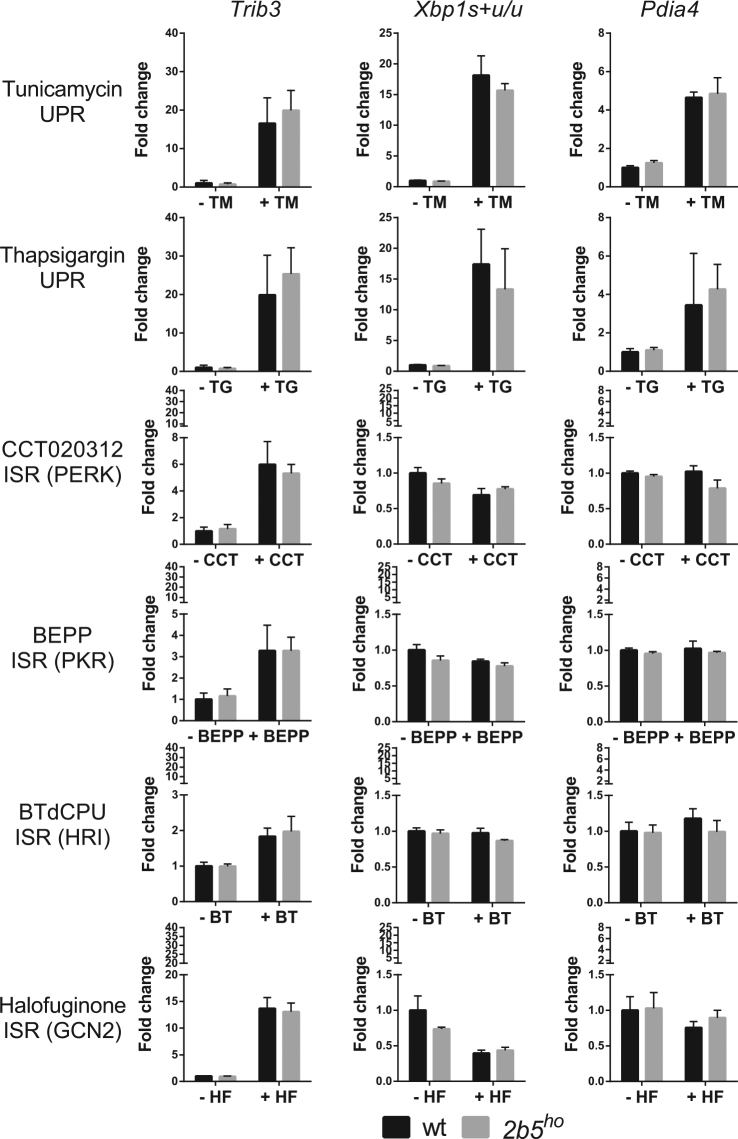
The UPR and ISR markers are similarly induced in wt and *2b5*^*ho*^ astrocytes upon short-term stress induction. UPR was induced by ER stressors tunicamycin (TM) or thapsigargin (TG). ISR was induced by treating cells with CCT020312 (CCT), BEPP, BTdCPU (BT) or halofuginone (HF) for 4 h. The kinases activated by ISR-inducing compounds are indicated (PERK, PKR, HRI or GCN2). The relative expression of mRNA markers of the UPR, *Trib3*, *Xbp1s* + *u/u and Pdia4*, was measured by qPCR after treatment. Values are fold change relative to vehicle-treated wt astrocytes. Graphs show average + SD (n = 3). P-values are shown in supplementary Table 1. *Trib3*, *Xbp1s* + *u/u* and *Pdia4* are significantly increased by tunicamycin and thapsigargin while only *Trib3* is significantly increased by CCT020312, BEPP, BTdCPU or halofuginone indicating ISR specificity. The increase is similar for wt and *2b5*^*ho*^ astrocyte cultures.

To assess whether *2b5*^*ho*^ primary astrocytes are hypersensitive to ISR activation, we cultured astrocytes in the presence of thapsigargin or tunicamycin. These compounds lead to inhibition of eIF2B activity via phosphorylation of eIF2α at Ser51 by PERK (ISR-activating kinase) and activate the ATF6 and IRE1α branches of the UPR. We subjected wt and *2b5*^*ho*^ astrocytes to either compound for a short interval (4 h). We assessed UPR activation by quantifying *Trib3*, *XBP1s* and *Xbp1u*, and *Pdia4* mRNAs that are specifically induced by transcription factors ATF4, XBP1s and ATF6-c respectively. The induction of *Trib3*, *Xbp1s* and *Pdia4* expression was substantial and significant but did not differ between wt and *2b5*^*ho*^ cells (Fig. 1). One could argue that tunicamycin and thapsigargin are not suitable for assessing the ISR in cells with mutated eIF2B. Both compounds activate the ATF6 and IRE1α branches of the UPR, each leading to activation of fairly distinct transcription programs^25,36^. It cannot formally be excluded that crosstalk between the three branches might mask an abnormal response to eIF2α phosphorylation in *2b5*^*ho*^ astrocytes. To further address this issue, we investigated selective activation of the ISR in *2b5*^*ho*^ astrocytes using four additional compounds (CCT020312, BEPP, BTdCPU, halofuginone) that were recently described as ISR inducers^37–40^. We subjected astrocytes to these compounds and measured induction of UPR mRNA markers. Activation of the ATF6 and IRE1α branches of the UPR was not observed as judged from changes in *Xbp1s* + *u/u* and *Pdia4* (Fig. 1). Treatment with either of these compounds induced the ISR to differing degrees as judged from the induction of *Trib3* mRNA, which ranged from 2-fold for BTdCPU to 15-fold for halofuginone (Fig. 1). Thapsigargin and tunicamycin induced *Trib3* mRNA expression approximately 20-fold (Fig. 1). Yet the ATF4-induced transcription did not differ between wt and *2b5*^*ho*^ (Fig. 1). We chose *Trib3* mRNA induction as a robust and sensitive marker for the integrated stress response as assessment of *Ddit3* mRNA expression was less sensitive and ATF4 protein induction more variable in our hands (Supplementary Fig. S1 and Supplementary Fig. S2). Still, differences between wt and *2b5*^*ho*^ astrocytes were not observed for any of the stress markers (Fig. 1, Supplementary Fig. S1 and Supplementary Fig. S2). These data suggest that activation of neither the ISR nor UPR was significantly affected by the Arg191His mutation in eIF2Bε.

**Figure 2:**
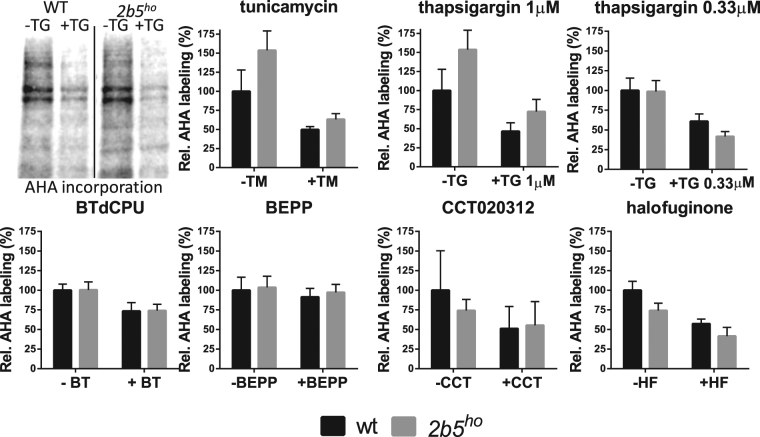
Protein synthesis rate is similarly reduced in wt and *2b5*^*ho*^ astrocytes in response to short-term UPR or ISR induction. Astrocytes were labelled with AHA for 2 h before cell lysis and AHA incorporation was determined as measure for the protein synthesis rate. Astrocytes were treated with ER stress inducers tunicamycin (TM), 1 μM thapsigargin (TG) or 0.33 μM thapsigargin or with ISR activators BTdCPU, BEPP, CCT020312, halofuginone for 4 h. Graphs show average + SD (n = 3–5). P-values are shown in supplementary Table 1. AHA incorporation decreased significantly upon TM, TG, BTdCPU or halofuginone treatment. The decrease is similar for wt and *2b5*^*ho*^ astrocyte cultures.

We next investigated the inhibition of protein synthesis in response to ISR. We subjected wt and *2b5*^*ho*^ astrocytes to either of the six ISR-inducing compounds and measured protein synthesis for a two-hour pulse using an azidohomoalanine (AHA) incorporation assay (Fig. 2). This sensitive and robust protocol relies on replacing the amino acid methionine in the culture medium with its analogue AHA^41^. AHA-incorporation was measured for 2 h in the presence or absence of tunicamycin or thapsigargin. Each treatment inhibited protein synthesis by approximately 50%, indicating that protein synthesis in wt and *2b5*^*ho*^ astrocytes was inhibited similarly (Fig. 2). The induction of the ISR by tunicamycin and thapsigargin was quite pronounced and perhaps too strong to detect possibly subtle differences in *2b5*^*ho*^ astrocytes. We thus repeated the experiment with a lower concentration of thapsigargin (0.33 μM) (Fig. 2). Protein synthesis was still significantly inhibited, but less than observed with previous concentration of thapsigargin (1 μM), confirming that the ISR activation by 0.33 μM thapsigargin was not maximal. The ISR-inducing compounds CCT020312 and halofuginone each inhibited protein synthesis by approximately 50%, similar to the inhibition observed with tunicamycin or thapsigargin (Fig. 2). The inhibition of protein synthesis was relatively small for BTdCPU and was not significant for BEPP (Fig. 2). Again, we did not observe a significant difference in response between wt and *2b5*^*ho*^ astrocytes.

### Wt and *2b5*^ho^ astrocytes respond similarly to long term UPR induction and recovery

**Figure 3:**
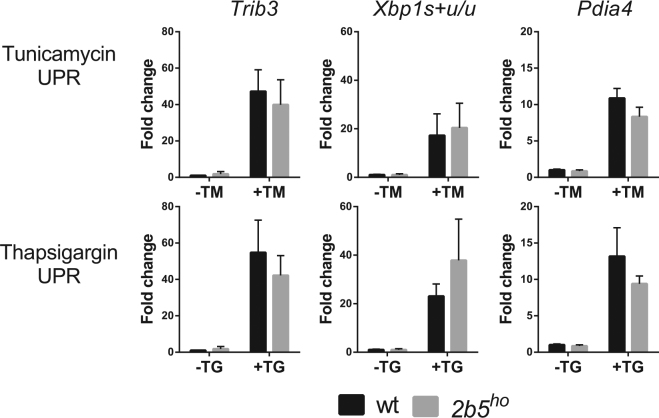
The UPR markers are similarly induced in wt and *2b5*^*ho*^ astrocytes upon long-term stress induction. UPR was induced by ER stressors tunicamycin (TM) or thapsigargin (TG) for 16 h. The relative expression of UPR mRNA markers *Trib3*, *Xbp1s* + *u/u and Pdia4* was measured by qPCR. Values are fold change relative to vehicle-treated wt astrocytes. Graphs show average + SD (n = 3). P-values are shown in supplementary Table 1. All markers are significantly increased after tunicamycin or thapsigargin treatment. The increase is similar for wt and *2b5*^*ho*^ astrocyte cultures.

So far we have investigated ISR and UPR induction in primary astrocytes over a relatively short interval. Possibly, *2b5*^*ho*^ primary astrocytes show differences in ISR activation when the exposure to stress is extended. This proposed effect would have been missed in the previous experiments. We extended the exposure to the selected stressors to 16 h and assessed mRNA expression (Fig. 3 and Supplementary Fig. S3). Both treatments induced significant UPR mRNA expression (Supplementary Table 1). We did not find consistent differences in stress response between wt and *2b5*^*ho*^ astrocytes. The response in *2b5*^*ho*^ astrocytes appeared to be normal at both short and long times.

**Figure 4:**
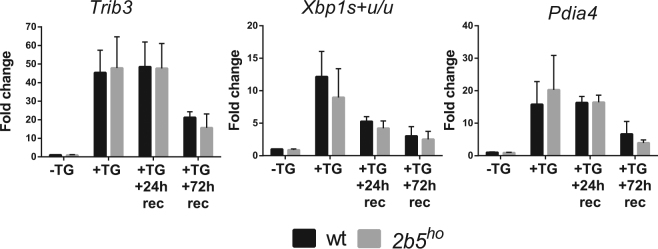
The UPR recovers similarly in wt and *2b5*^*ho*^ astrocytes. UPR was induced by ER stressor thapsigargin (TG) for 24 h. TG was subsequently removed and cells were left to recover for 24 (+TG + 24 h rec) or 72 h (+TG + 72 h rec). The relative expression of UPR mRNA markers *Trib3*, *Xbp1s* + *u/u and Pdia4* was measured at indicated times by qPCR. Values are the fold change relative to vehicle-treated wt astrocytes (−TG, 24 h). Graphs show average + SD (n = 3). P-values are shown in supplementary Table 1. *Trib3*, *Xbp1s* + *u/u* and *Pdia4* are is significantly increased at all time points. The increase is similar for wt and *2b5*^*ho*^ astrocyte cultures.

Alternatively, *2b5*^*ho*^ astrocytes may recover abnormally from stress. We further increased the stress duration to 24 h with thapsigargin in wt and *2b5*^*ho*^ astrocytes. We then replaced the culture medium and omitted thapsigargin. The cells were then left to recover for 24 h or 72 h. The UPR marker mRNAs of all three branches decreased over time during stress recovery (Fig. 4, Supplementary Fig. S3). *Trib3*, *Ddit3*, *Xbp1s* + *u*, *Xbp1u* and *Pdia4* mRNA expression were similar in wt and *2b5*^*ho*^ astrocytes at all measured time points. Together these results indicate that the induction of the UPR or subsequent recovery from it do not differ between wt and *2b5*^*ho*^ astrocytes at the mRNA level (Fig. 4).

### Wt and *2b5*^*ho*^ astrocytes respond to ISR and UPR induction at the level of cell viability

**Figure 5:**
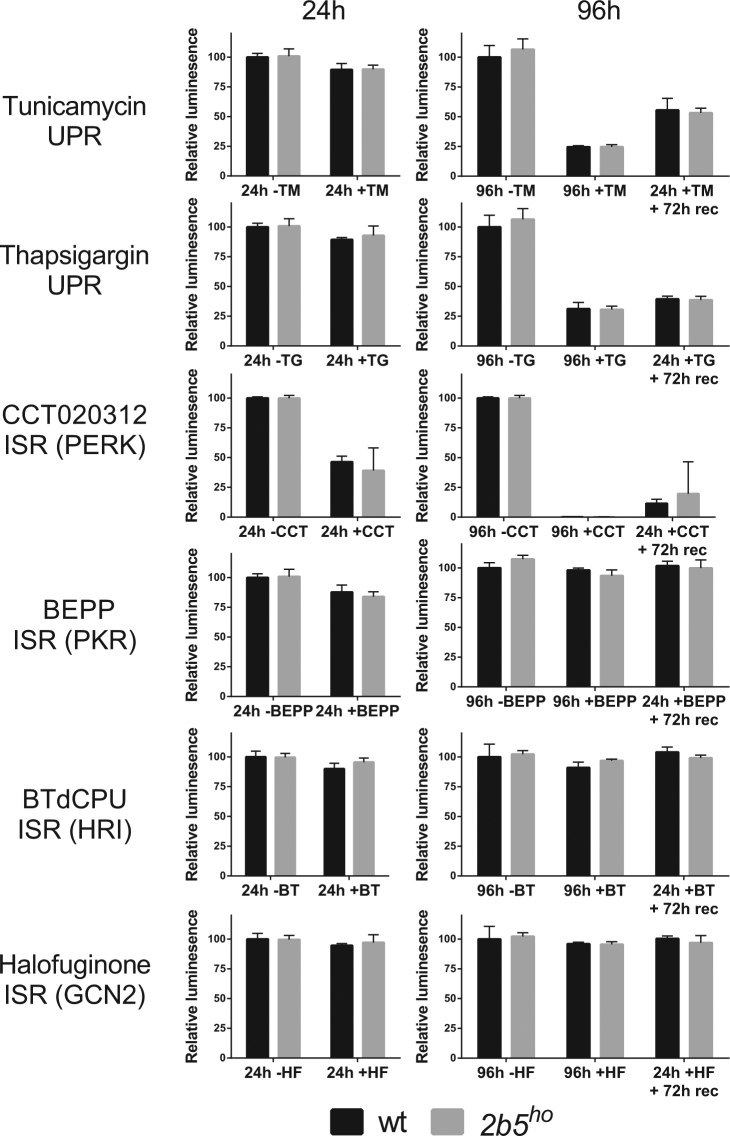
Cell viability upon UPR or ISR induction is not differentially affected in wt and *2b5*^*ho*^ astrocytes. UPR was induced by ER stressors tunicamycin (TM) or thapsigargin (TG). ISR was induced by CCT020312 (CCT), BEPP, BTdCPU (BT), or halofuginone (HF). The kinases activated by ISR compounds are indicated (PERK, PKR, HRI or GCN2). Cell viability was measured 24 and 96 h after stress induction without and with recovery after 24 h. Values are relative cell viability compared to vehicle-treated cultures. Graphs show average + SD (n = 3; CCT in wt, n = 2). P-values are shown in supplementary Table 1. Tunicamycin and CCT020312 significantly reduces cell viability under all test conditions. BEPP only significantly reduces the cell viability after 24 h while thapsigargin significantly reduced cell viability only after 96 h. The cell viability was similar for wt and *2b5*^*ho*^ astrocyte cultures.

**Figure 6:**
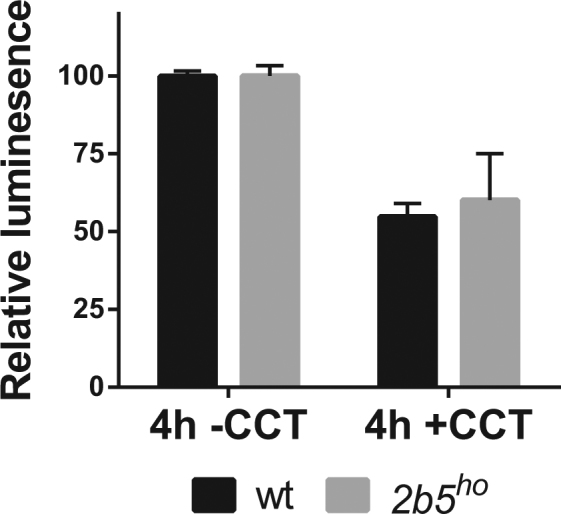
Cell viability upon short-term CCT020312 treatment is not differentially affected between wt and *2b5*^*ho*^ astrocytes. Cell viability was measured after 4 h of CCT020312 (CTT) treatment. Values are relative cell viability compared to vehicle-treated cultures. Graphs show average + SD (n = 3, wt CCT n = 2). P-values are shown in supplementary Table 1. Although cell viability was significantly reduced, no significant difference in response was observed between wt and *2b5*^*ho*^ astrocyte cultures.

After investigation of the ISR and UPR on mRNA as well as protein synthesis level, we interrogated the cellular effects in response to stress. We assessed viability of both wt and *2b5*^*ho*^ astrocytes in response to long term exposure to the various compounds (i.e. 24 h and 96 h) as well as recovery (24 h stressor and subsequent 72 h recovery). None of the compounds significantly reduced cell viability at 24 h with the exception of CCT020312 (Fig. 5). After 96 h cell viability was compromised by PERK activators tunicamycin, thapsigargin and CCT020312, but not by the ISR activators that mediate the response via PKR, GCN2 or HRI. To assess stress recovery the stressor was removed after 24 h and cell viability was determined after 96 h. The PERK activators negatively impacted on cell viability under all conditions, whereas the other compounds did not. Still, the cell viability was similar between wt and *2b5*^*ho*^ astrocytes under all tested conditions. As CCT020312 already strongly reduced cell viability at 24 h (approximately 50%), we tested cell viability at 4 h and found that also at this early time-point it was reduced by approximately 40% (Fig. 6). Again, this assay did not detect a different cellular response to UPR or ISR induction in *2b5*^*ho*^ astrocytes, in line with the results obtained thus far. The reduction in cell viability varied between the different stressors but, importantly, was similar for wt and *2b5*^*ho*^ astrocytes for any given stress.

## Discussion

We previously found that astrocytes are likely the cells that are primarily affected cells in VWM^12^. At the start of the study, we selected astrocyte cultures for testing ISR or UPR hypersensitivity, as this system allows multiple manipulations to modulate eIF2B activity. We focused on the *2b5*^*ho*^ astrocytes as this mouse model showed a more severe phenotype than the *2b4*^*ho*^ model, which is homozygous for the eIF2Bδ mutation Arg484Trp^12^. The *2b5*^*ho*^ astrocytes have recently been reported to reveal differences in metabolite accumulation and in synthesis rates for 80 proteins. The function of some of these eIF2Bε^Arg191His^-regulated proteins is linked to astrocyte differentiation, which is known to be disturbed in VWM^35^. The eIF2Bε Arg195His mutation leads to a severe neurological phenotype in patients^14^. Recombinant human eIF2B with the Arg195His mutation in eIF2Bε showed a significantly decreased enzymatic activity to approximately 50% of wt eIF2B activity^42^, indicating that the *2b5*^*ho*^ mouse model homozygous for the equivalent Arg191His mutation is also intrinsically affected in eIF2B activity.

We now report that the *2b5*^*ho*^ astrocytes do not show signs of expected consequences of reduced eIF2B activity: they grew with similar rates and incorporated the AHA label with similar efficiency as wt astrocytes, indicative of normal protein synthesis rates (Fig. 2A). Surprisingly, the ISR and UPR in astrocytes were not significantly affected by the Arg191His mutation in eIF2Bε. Together these observations suggest that in primary astrocyte cultures eIF2B activity is not rate limiting for protein synthesis under normal and stress conditions. A recent study reached a similar conclusion: Chinese hamster ovary cells homozygous for eIF2Bδ mutations Ala391Asp or Arg483Trp did not show altered protein synthesis or UPR induction, although the mutations reduced the eIF2B activity in the biochemical assay more than 50%^33^. Alternatively, it is possible that reduced eIF2B activity in *2b5*^*ho*^ astrocytes is compensated by a yet to be determined mechanism that is not evident in biochemical assays.

In this study, we aimed to test the hypothesis that VWM mouse astrocytes respond hypersensitively to ISR and UPR activation compared to wt astrocytes. We induced the ISR with various compounds that activate eIF2α kinases and thus impair eIF2B function. We assessed the stress pathways at different levels: mRNA induction, protein synthesis rate and cell viability. The response differed in magnitude among the compounds tested. Tunicamycin and thapsigargin induced a relatively strong response and the PKR activator BEPP a modest response (compare y axes of Figs 1 and 2). The difference in ISR induction amongst the various activators is an interesting observation. However, at the moment we cannot discriminate whether these compounds are taken up by cells with differing efficiency or if they differ in their effectiveness in activating the relevant specific eIF2 kinases. Alternatively, the specific kinases in mouse primary astrocytes may differ in expression or specific activity. The latter could be true for PERK, which is activated efficiently in response to thapsigargin, tunicamycin or CCT020312, suggesting a relatively high activity. Also, the ISR activators tested did not significantly activate the ATF6- and XBP1s-driven transcription programs, confirming that they do not activate a full UPR (Fig. 1). Together our data indicate that under low, high, long, short UPR or ISR induction an abnormal response in *2b5*^*ho*^ primary astrocytes is not evident.

In general, viability of the mouse astrocyte cultures was not markedly affected by the stress inducers, and no differences were noted between wt and mutant cells. With the exception of CCT020312, none of the compounds greatly reduced astrocyte viability after exposure interval of 24 h. Other cell types such as neuronal cell lines or primary rat cardiac myocytes show a marked reduction in cell viability upon 24 h thapsigargin or tunicamycin treatment^43,44^. In support of our findings UPR activation by 24 h thapsigargin did not lead to reduced cell viability in astrocytes derived from embryonic mice^45^. BEPP grossly reduced the viability of mouse embryonic fibroblast after 72 h, while halofuginone did not affect the viability of human fibroblasts or osteosarcoma cells^37,46,47^. These results suggest that the cell type and growth conditions highly influence the response to UPR or ISR activation. CCT020312 was initially identified in a screen searching for a cell cycle arrest factor and was subsequently found to activate PERK as underlying mechanism^40^. This is an interesting finding: activation of PERK reduced cell viability most in adult murine astrocytes compared to the other stressors. However, under these circumstances no differences were observed between wt and *2b5*^*ho*^ primary astrocytes.

In VWM patients’ brains the ISR/UPR is activated in glia^23,28^. It is not known whether the activated UPR in patients’ glia is a hallmark of a protective reaction to the disease or if it contributes to neuropathology or clinical signs in patients. Patients show periods of rapid deterioration as a consequence of physical stresses. Possibly, during these events the ISR or UPR in glia become hyperactivated as a consequence of the eIF2B mutations. We cannot rule out that a minor difference in stress responses in *2b5*^*ho*^ primary astrocytes was missed in the current study, despite the severity of the eIF2Bε Arg191His mutation in patients. Nonetheless, the differences in ATF4 and CHOP protein expression were readily detected in VWM patients’ brain tissue by qPCR and Western blotting^23,24^ and it is reasonable to conclude that these differences in expression were not recapitulated in the current cell system. On the basis of the current findings, we cautiously propose that the eIF2Bε Arg191His mutation does not intrinsically affect the kinetics of the ISR or UPR in cultured astrocytes.

Probably, the mutant mouse astrocyte cultures are not representative of the astrocytes in patients’ or mutant mouse brains. Both astrocyte morphology and GFAPδ-expression have been reported to be abnormal in patient and *2b5*^*ho*^ mouse brain^12^. These features are not recapitulated in the *2b5*^*ho*^ astrocyte cultures. We should point out that *2b5*^*ho*^ primary astrocytes from E18 mice were deficient in supporting oligodendrocyte maturation *in vitro*, despite similar morphology and GFAP-expression when compared to wt astrocytes^12^. Human astrocyte cultures from VWM patients are a very limited resource and therefore we cannot address whether human astrocytes would be a superior model to the murine astrocytes. We suspect that these cells share the problem of not fully recapitulating the phenotype of cells in intact tissue. Responses to febrile stress or tissue damage in patients’ brain may also involve ISR- or UPR-independent mechanisms that are not yet fully understood. The elevation of ISR/UPR markers in VWM brain may reflect the interplay between additional factors, including ones that are not intrinsic to astrocytes.

## Materials and Methods

### Animals

All experiments were carried out under the Dutch/European law and with approval of the local animal care and use committee of the VU University of Amsterdam (permit number FGA 11-05, FGA 14-04). Wt and *2b5*^*ho*^ mice were used^12^. All animals were weaned at P21 and subsequently had *ad libitum* access to food and water. The mice were housed with a 12 h light/dark cycle. Mice were genotyped as described^12^.

### Astrocyte culture

Astrocytes were isolated from gender-matched 4-month-old wt and *2b5*^*ho*^ mice in parallel. Mice were sacrificed by cervical dislocation. Brains were taken out; the olfactory bulb, cerebellum and cortex were removed. Astrocytes were isolated from the remaining structures, as described^35^. Cells were grown at 37 °C and 5% CO_2_. After 2 passages we generated approximately 10^7^ astrocytes per genotype. Every experiment was replicated in independent cultures derived from different mice (number of experiments is indicated in figure legend as e.g. n = 3).

### Treatments with chemical stressors

Cells were stressed with various compounds for indicated times. At the start of treatment, we replaced the culture medium with DMEM/F12 medium supplemented with UPR activators tunicamycin (2.5 µg/ml; Sigma) and thapsigargin (0.33 and 1 µM; Sigma) or GCN2 activator halofuginone (10 nM; Cayman Chemical Company)^39^, HRI activator BTdCPU (KM09748SC) (6 µM; Thermofisher)^38^, PKR activator BEPP (10 µM; Sigma)^37^ or PERK activator CCT020312 (10 µM; Millipore) which specifically activate the ISR^40^. All compounds were dissolved in DMSO. Control cultures were simultaneously treated with equal amounts of DMSO (vehicle control).

### RNA isolation, cDNA synthesis and qPCR

Astrocytes were plated in 10 cm dishes (~750,000 cells/dish). The astrocytes were cultured until 80% confluent. Cells were subsequently treated with the indicated stressors for the indicated time. Cells were washed with cold PBS (Gibco) and collected in TRIzol^TM^ Reagent (Invitrogen). RNA was isolated as described^35^.

RNA quality and quantity was determined by measuring the A260 and A280 (Nanodrop 2000, Thermo Scientific). cDNA was synthesized and mRNA levels were measured as described^35^. For each 10 µl qPCR sample a mixture of SYBR green (Roche), primers (1 pmol/µl) and cDNA (0.1 µl) was used. The primers used were validated (Supplementary Fig. S4) and are listed in Table 1. *Gapdh* mRNA was used as reference for normalization. Sequence analyses were performed on cDNA from 42 samples from wt and *2b5*^*ho*^ cells to confirm the absence or presence of the c.572 G > A, p.Arg191His mutation in *Eif2b5* (Supplementary Fig. S5).

**Table 1:** Oligonucleotide primers.

Gene name	Forward (5′ → 3′)	Reverse (5′ → 3′)
*Gapdh*	GTGCTGAGTATGTCGTGGAG	TCGTGGTTCACACCCATCAC
*Ddit3*	CTGGTATGAGGATCTGCAGG	TTGATTCTTCCTCTTCGTTTCC
*Trib3*	TGTCTTCAGCAACTGTGAGAGGACGAAG	GTAGGATGGCCGGGAGCTGAGTATC
*Xbp1u*	GCAGCACTCAGACTATGTG	CCAACTTGTCCAGAATGCCC
*Xbp1s* + *u*	TCCGCAGCAGGTGCAG	CCAACTTGTCCAGAATGCCC
*Pdia4*	GGTCATCATTGGGCTCTTTCAG	GGAACTTGGCTATTTCAGGGC

### Determination of protein synthesis rates

Astrocytes were plated in 6 cm dishes (~250,000 cells/ dish) and were cultured until 80–90% confluency. Indicated stressors were added for 4 h. Protein synthesis was determined with AHA incorporation, as described^35,41^. AHA (Bachem) was added for the last two hours of the experiment to allow sufficient AHA incorporation into newly synthesized proteins. The newly synthesized proteins were quantified visualized as described^35^. The amount of staining is corrected for the total protein load determined by Gel Doc™ EZ System (Biorad).

### Cell viability

Astrocytes were plated in ½ area 96 well plates (~5,000 cells/well; Greiner Bio-one). Cells were cultured with the indicated compounds for the indicated time. Cell viability was measured based on the quantification of intracellular ATP levels as a measure for metabolically active cells. The assay is performed according to manufacturer’s instructions (Promega). In short, cells were kept at room temperature for 30 min. CellTiter-Glo (Promega) was added in the same volume as the culture medium. The plate was shaken for 2 min and left standing for 5 min before measuring the luminescence with a Victor2 (Perkin Elmer Life Sciences) as described^35^.

### Statistical analysis

The software program Factor was used to correct for differences between experiments but not between other conditions within experiments (genotype, treatments)^48^. For the qPCR experiments, all data points in one experiment (displayed in one graph) are divided by the average of data point for each mRNA in all wt DMSO. For the AHA incorporation experiments the average incorporation of all wt DMSO was set at 100%. The cell viability values depended heavily on cell number and therefore these experiments were normalized separately for wt and *2b5*^*ho*^. Statistical differences were determined using SPSS. We tested if the effect of treatment was different between wt and *2b5*^*ho*^ astrocytes (univariate analysis of variance, UNIANOVA, two-tailed).

### Data availability

The datasets generated and analysed during the current study are available from the corresponding author on reasonable request.

## Electronic supplementary material

Supplementary figures

Dataset 1
