# Supplementary material for: Adult mouse eIF2Bε Arg191His astrocytes display a normal integrated stress response in vitro
Source: Sci Rep. 2018 Feb 28;8:3773. doi: 10.1038/s41598-018-21885-x (PMC5830650; doi:10.1038/s41598-018-21885-x)
Supplement: Supplementary file 1 — Supplementary figures [file 41598_2018_21885_MOESM1_ESM.pdf]

Adult mouse eIF2Bε Arg191His astrocytes display a normal integrated stress response in vitro

Lisanne E. Wisse, Timo J. ter Braak, Malu-Clair van de Beek, Carola G.M. van Berkel, Joke Wortel, Vivi M. Heine, Chris G. Proud, Marjo S. van der Knaap and Truus E.M. Abbink

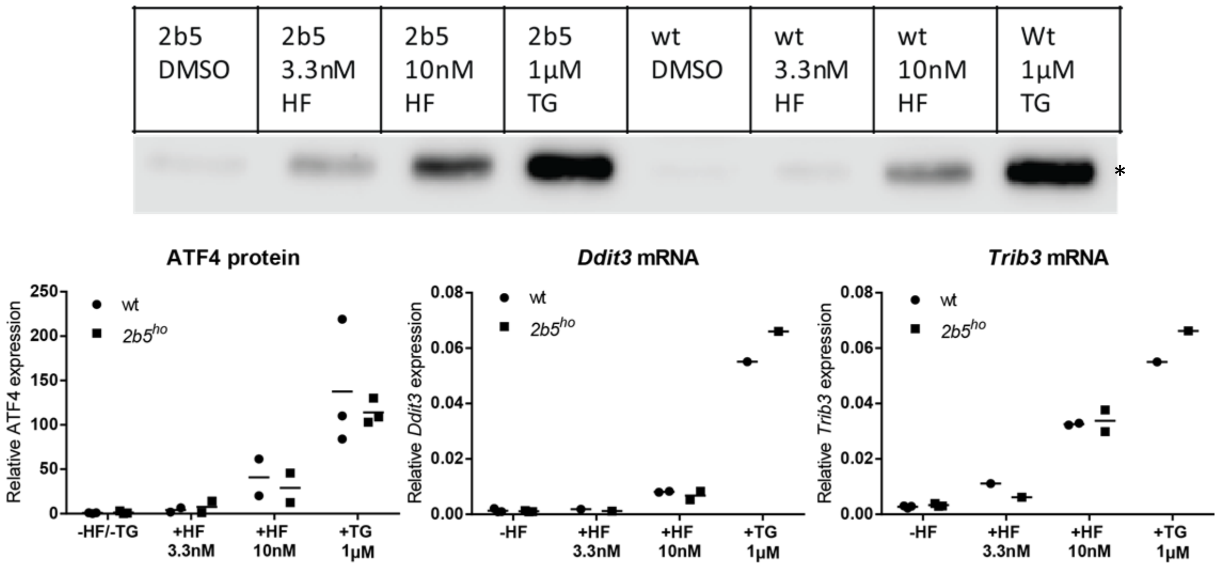

Supplementary Fig. S1: ATF4 protein expression in *2b5^{ho}* is similar to wt astrocytes after TG en HF treatment. Cultures of wt and *2b5^{ho}* astrocytes were treated with different concentrations of HF (3.3 and 10nM) and 1μM TG for 4 hours. HF/TG protein expression, HF mRNA expression and TG mRNA expression were performed with independent cell cultures. ATF4 protein expression was measured in isolated nuclear fractions (Ron protocol) using the ATF4 SC-200 antibody (indicated with *). The expression was quantified and corrected for total protein loading. The ATF4 levels after TG treatment are higher than after HF treatment indicating that HF treatment does not induce a maximal ATF4 expression. Also, 3.3nM HF induces a lower ATF4 protein expression than 10nM HF which is in line with our findings for *Ddit3* and *Trib3* mRNA levels obtained with qPCR. Western blot image is cropped around the ATF4 band (full blots are shown in raw data Fig 2).

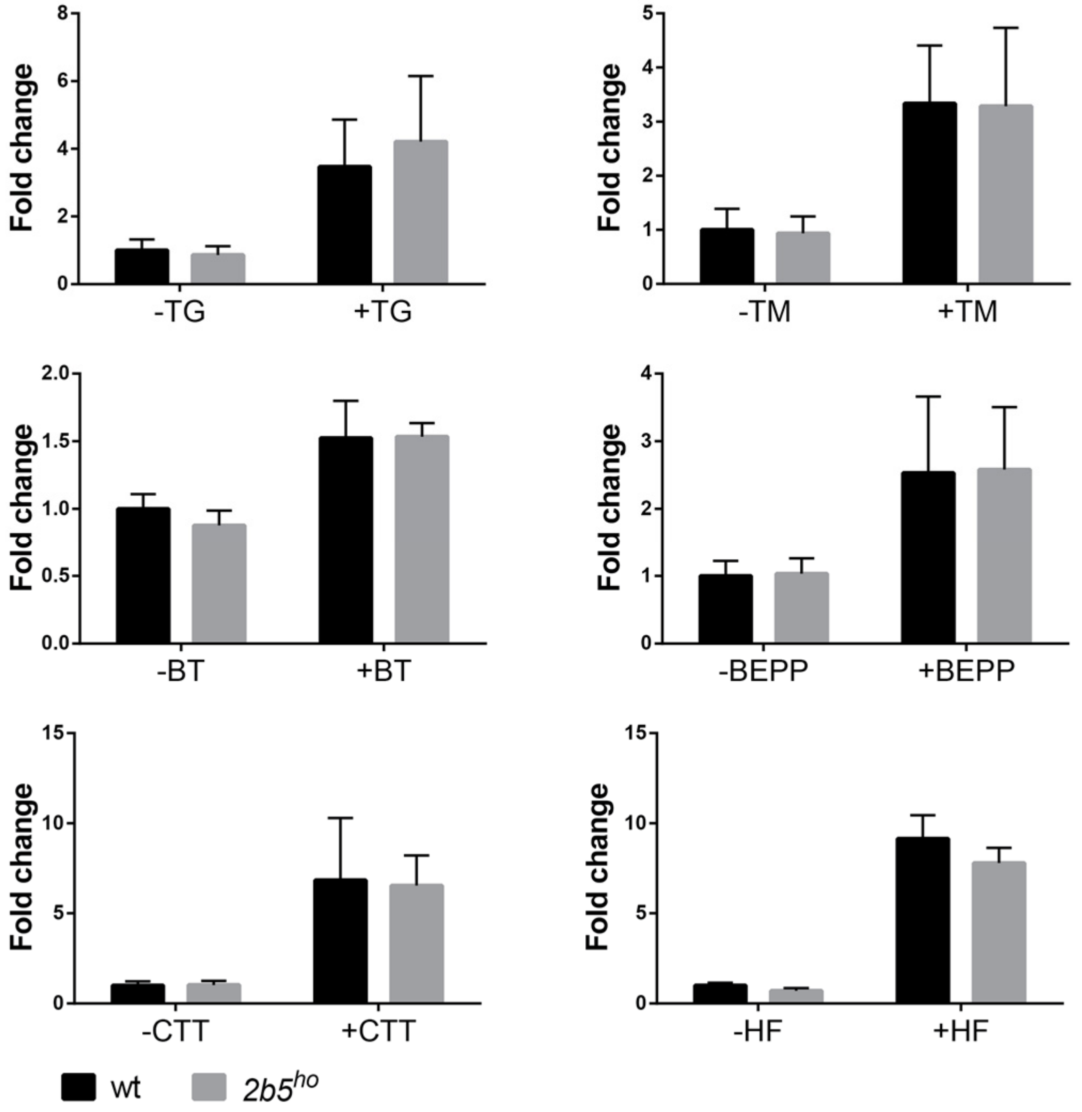

Supplementary Fig. S2. *Ddit3* mRNA expression is normal in *2b5^ho^* astrocytes after short-term UPR or ISR induction. UPR was induced by ER stressors tunicamycin (TM) or thapsigargin (TG). ISR was induced by CCT020312 (CCT), BEPP, BTdCPU (BT) or Halofuginone (HF) for 4 h. The relative expression of *Ddit3* mRNA was measured by qPCR. Values are fold change relative to vehicle-treated wt astrocytes. Graphs show average + SD (n=3). Differences between untreated and treated cells are significant at P<0.05, two way ANOVA (for BEPP treatment p=0.07). *Ddit3 is* significantly increased by all treatments. The increase is similar for wt and *2b5^ho^* astrocyte cultures.

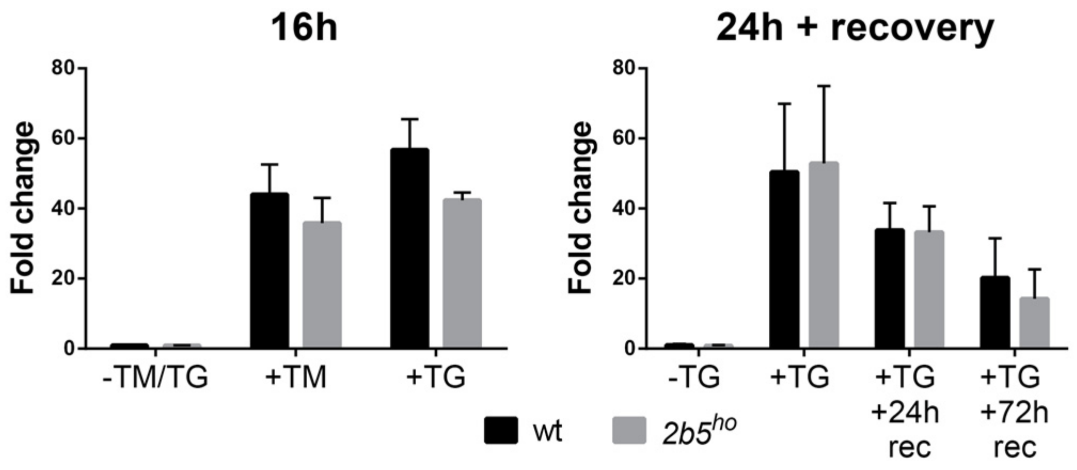

Supplementary Fig. S3. *Ddit3* mRNA expression is normal in *2b5*^{ho} astrocytes after long-term UPR induction and recovery. A. UPR was induced by tunicamycin (TM) or thapsigargin (TG) for 16 h. B. UPR was induced by ER stressor thapsigargin (TG) for 24 h. TG was subsequently removed and cells were left to recover for 24 (+TG +24 h rec) or 72 h (+TG +72 h rec). The relative expression of *Ddit3* mRNA was measured by qPCR and was significantly increased by TM or TG. Values are fold change relative to vehicle-treated wt astrocytes. P-values are shown in supplementary table 1. Graph shows average + SD (n=3).

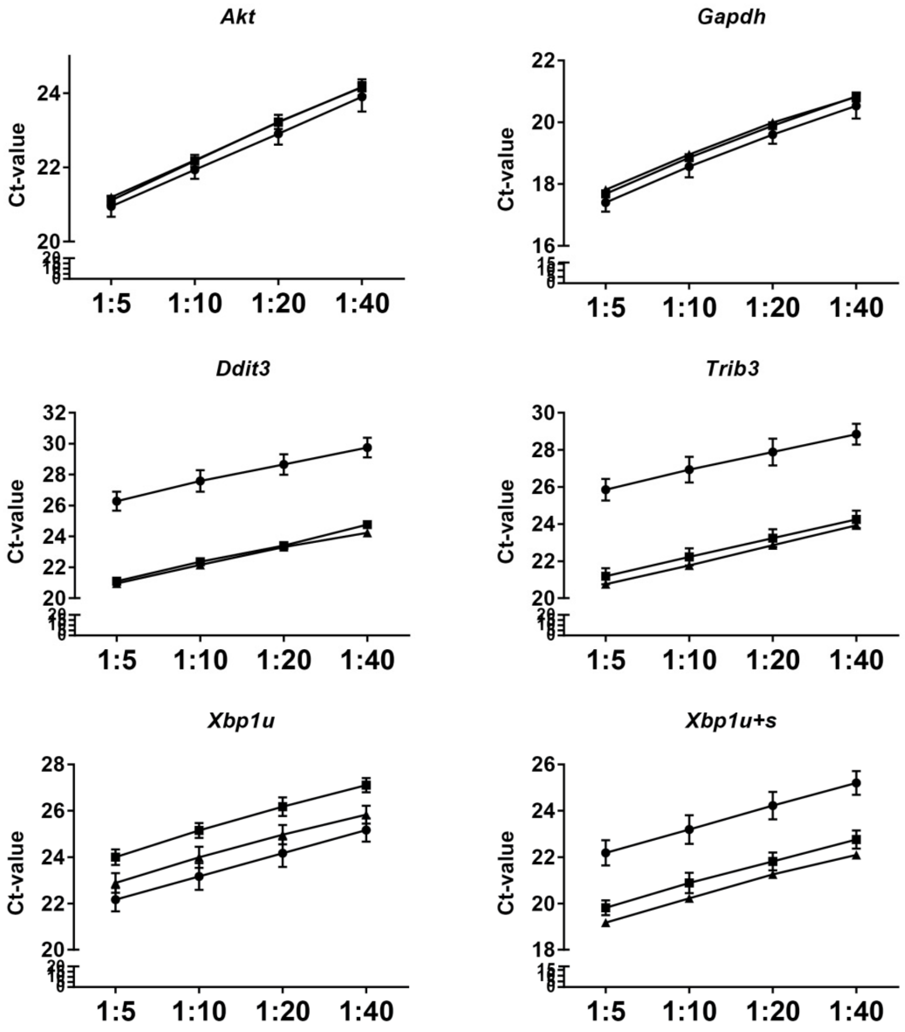

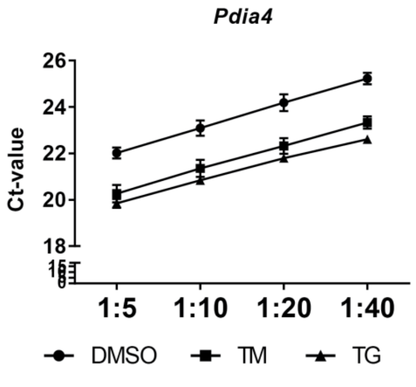

gene	primer efficiency
Akt	1.99
Gapdh	1.81
Ddit3	1.79
Trib3	1.97
Xbp1u	1.92
Xbp1s+u	1.97
Pdia4	1.96

Supplementary Fig. S4. Primer validation. The primers were validated using a dilution range of cDNA (1:5, 1:10, 1:20, 1:40) from wt and *2b5*^{ho} astrocyte cultures without treatment of treated with tunicamycin (TM) or thapsigargin (TG). The selected samples were used to validate that the primer efficiency was independent of the UPR marker expression. The primer efficiency was comparable for all tested samples and is summarized in the table.

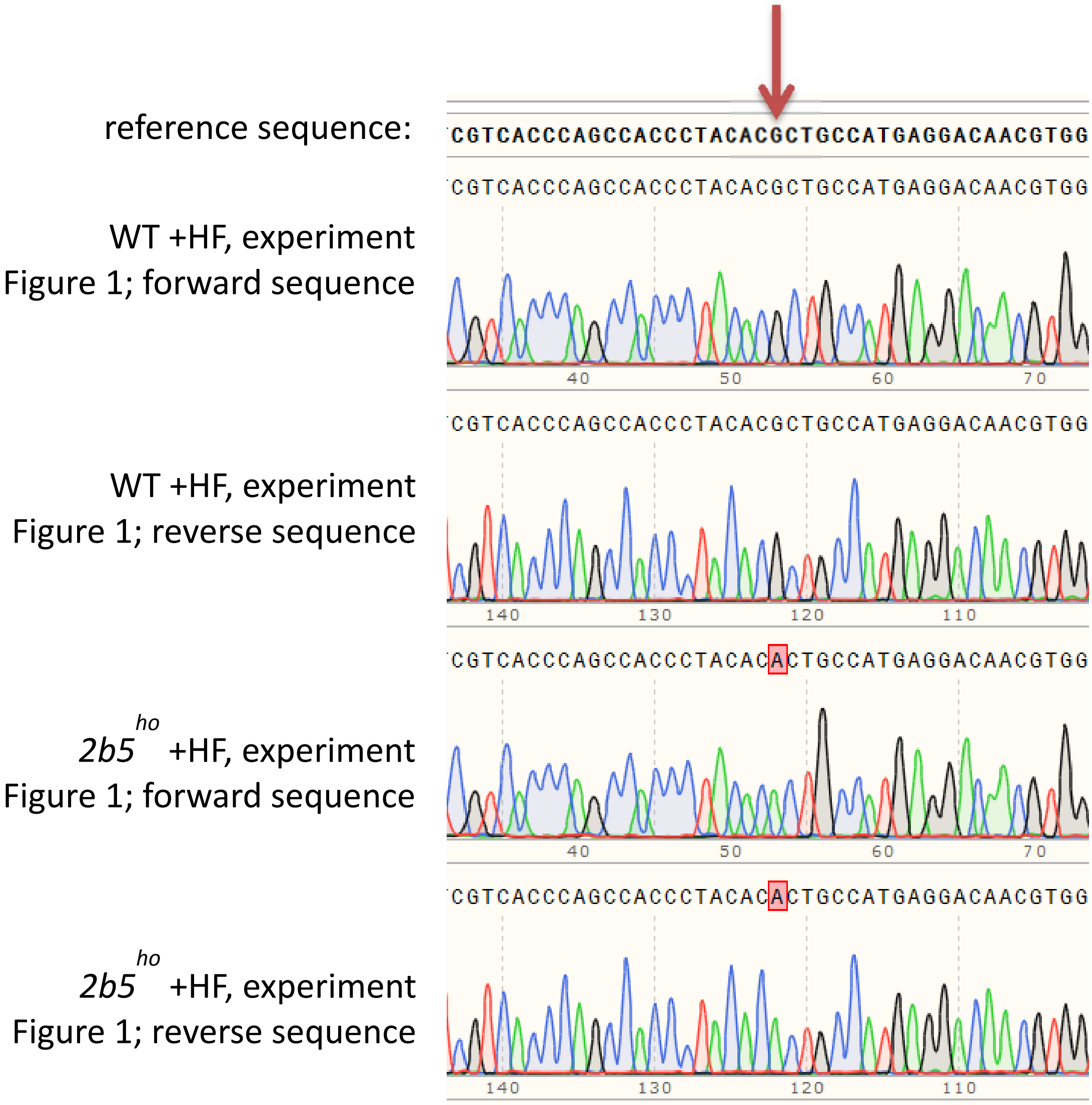

Supplementary Fig. S5. Aligned sequences of *Eif2b5* cDNA. Exon 4 of *Eif2b5* cDNA was amplified by polymerase chain reaction using primers O-8377 (5’-GAGGAACACAGGTTAAGAAGG-3’) and O-8378 (5’-GTGACAGTCCAACAAATCATATCG-3’) and analyzed by Sanger sequencing. Samples from WT and *2b*^{ho} animals were analyzed for 13 independent experiments. Representative electropherograms are shown for WT and mutant sequences (both forward and reverse reads). The reverse read is shown as reverse complement. The nucleotide position c.572 is indicated with a red arrow (*2b*^{ho} mice are homozygous for the c.572G>A, p.Arg191His mutation in *Eif2b5*).

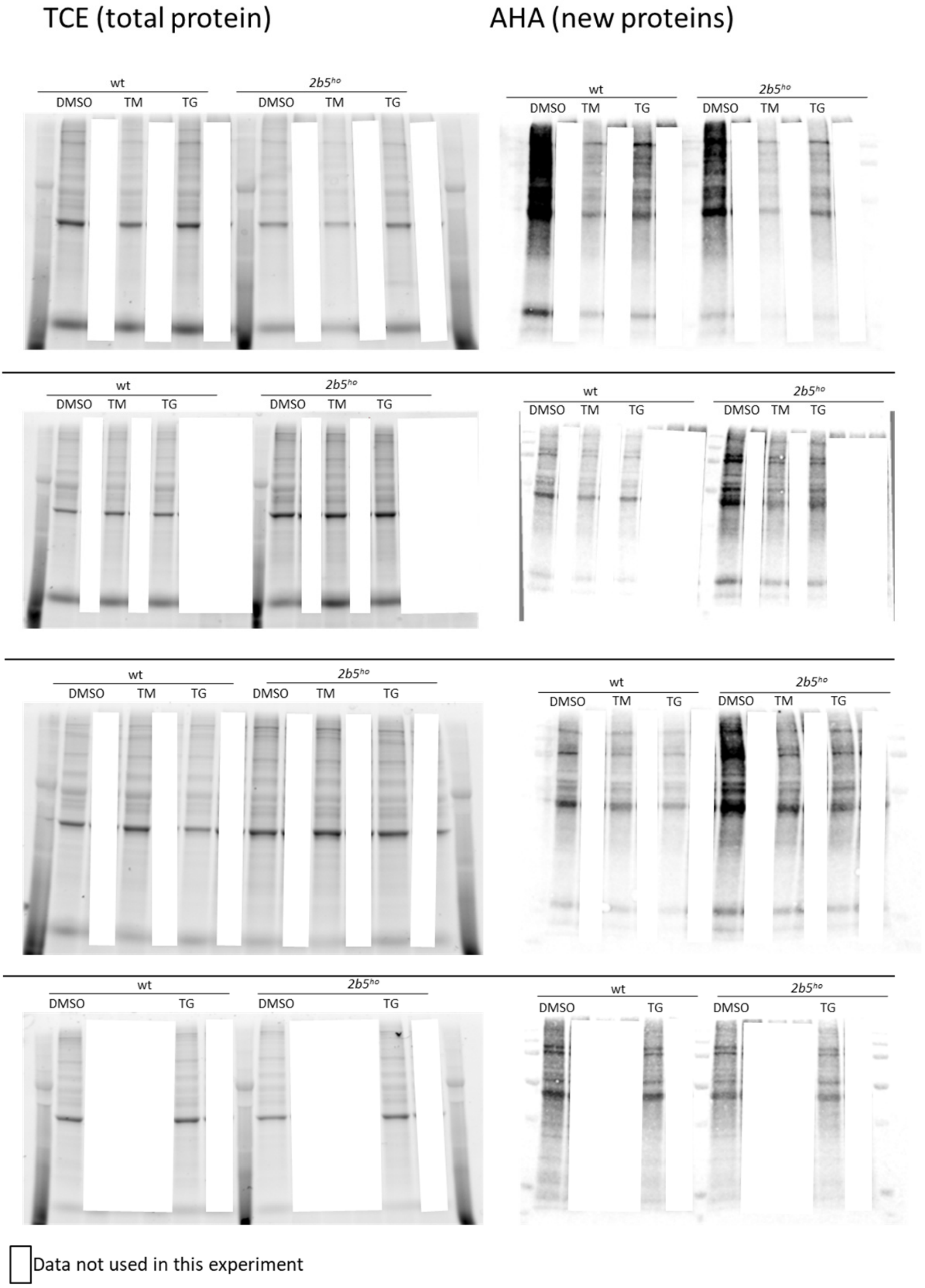

Overview of blots used for figure 2 (page 1 out of 4)

TCE (total protein)

AHA (new proteins)

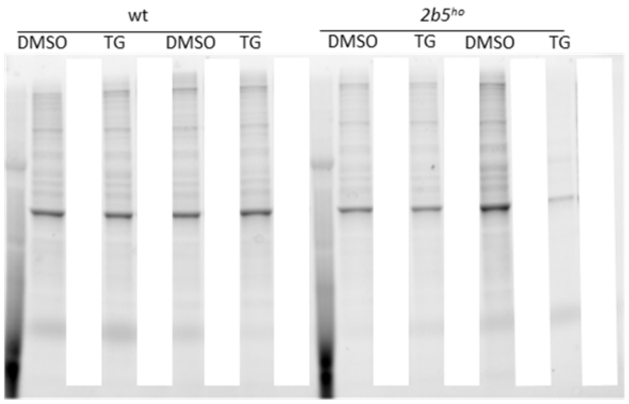

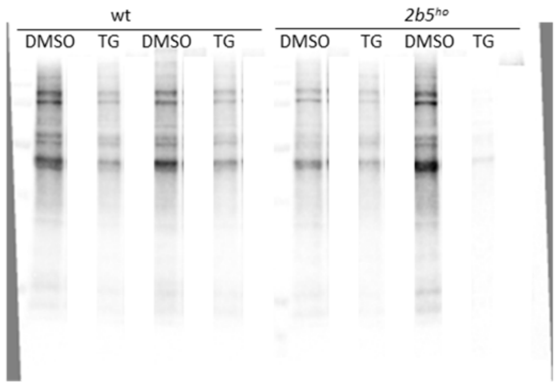

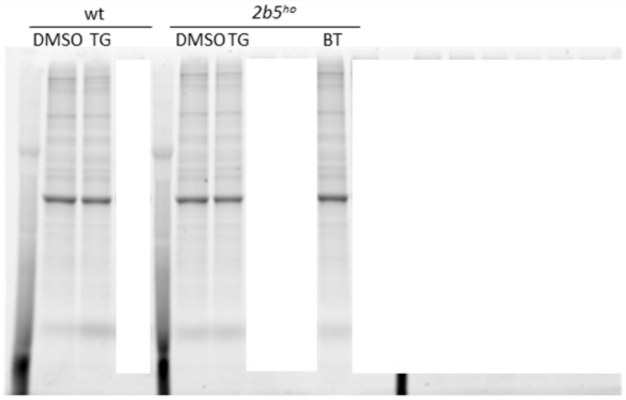

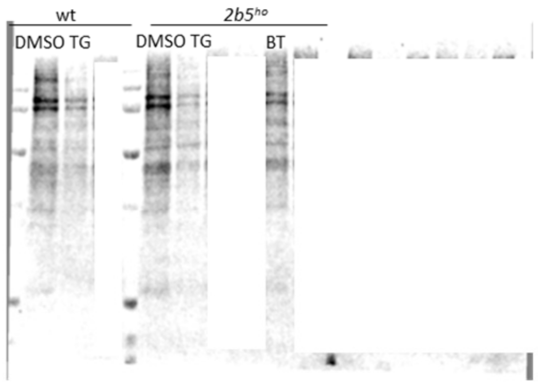

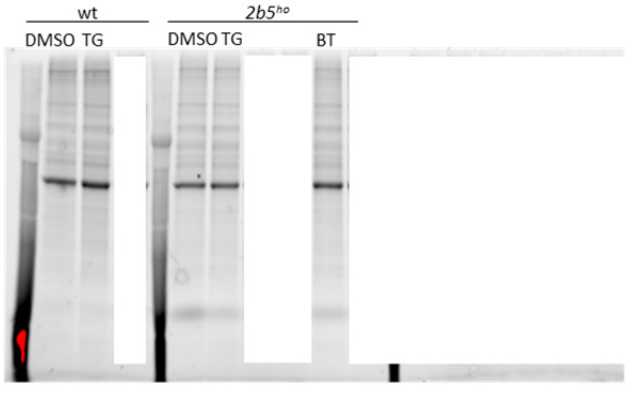

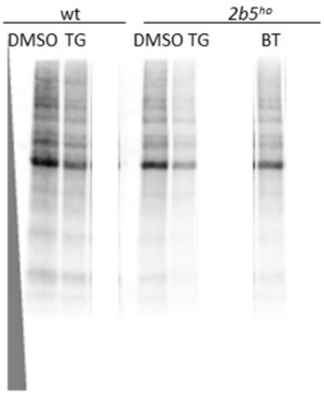

Data not used in this experiment

Overview of blots used for figure 2 (page 2 out of 4)

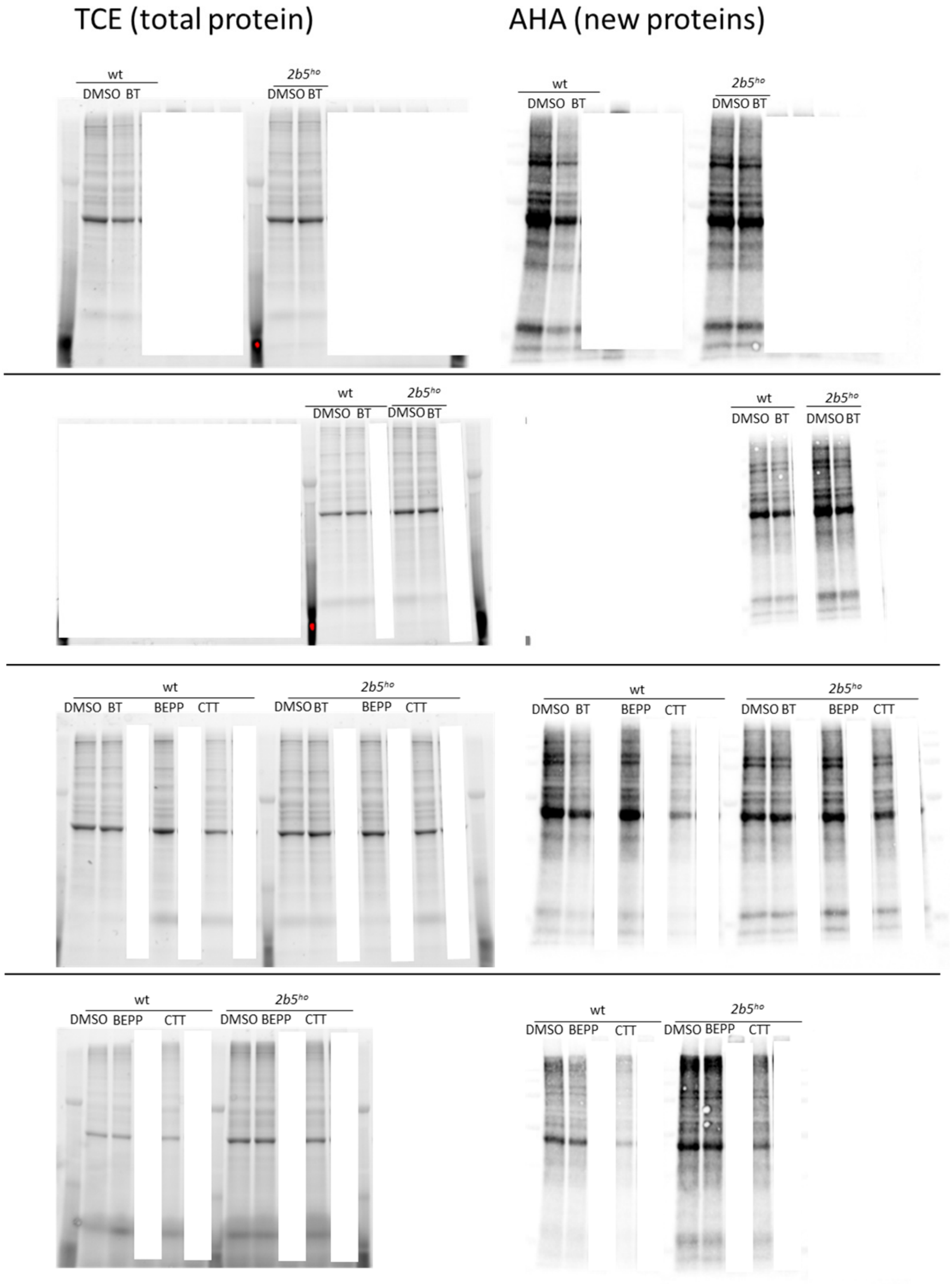

Data not used in this experiment

Overview of blots used for figure 2 (page 3 out of 4)

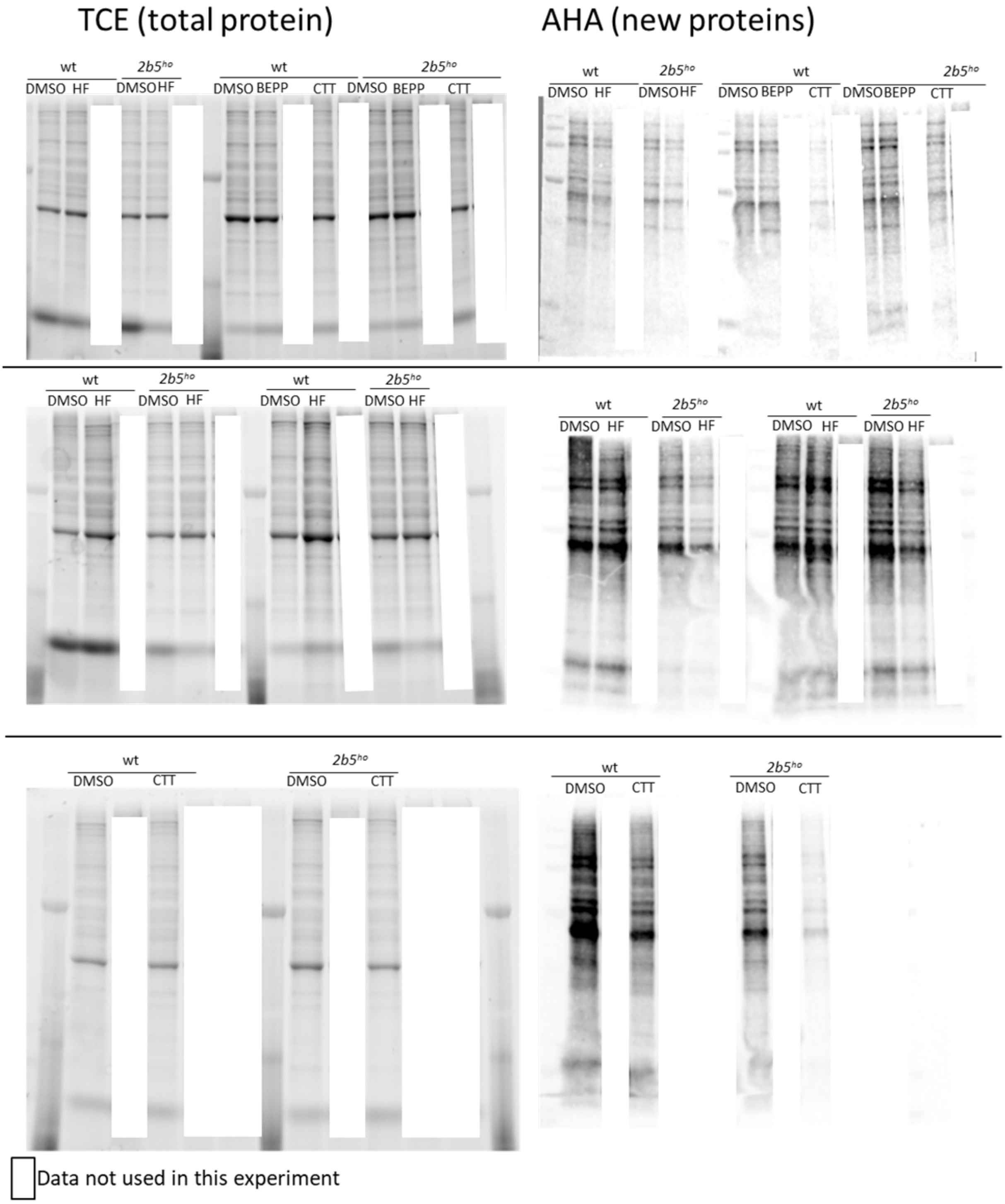

Overview of blots used for figure 2 (page 4 out of 4). Each panel contains a TCE stain (total protein) with the corresponding AHA stain (newly sythesized proteins). Full blots are shown. The whitened parts of the blots contain data that were not used in the experiments presented in this manuscript.

TCE (total protein)

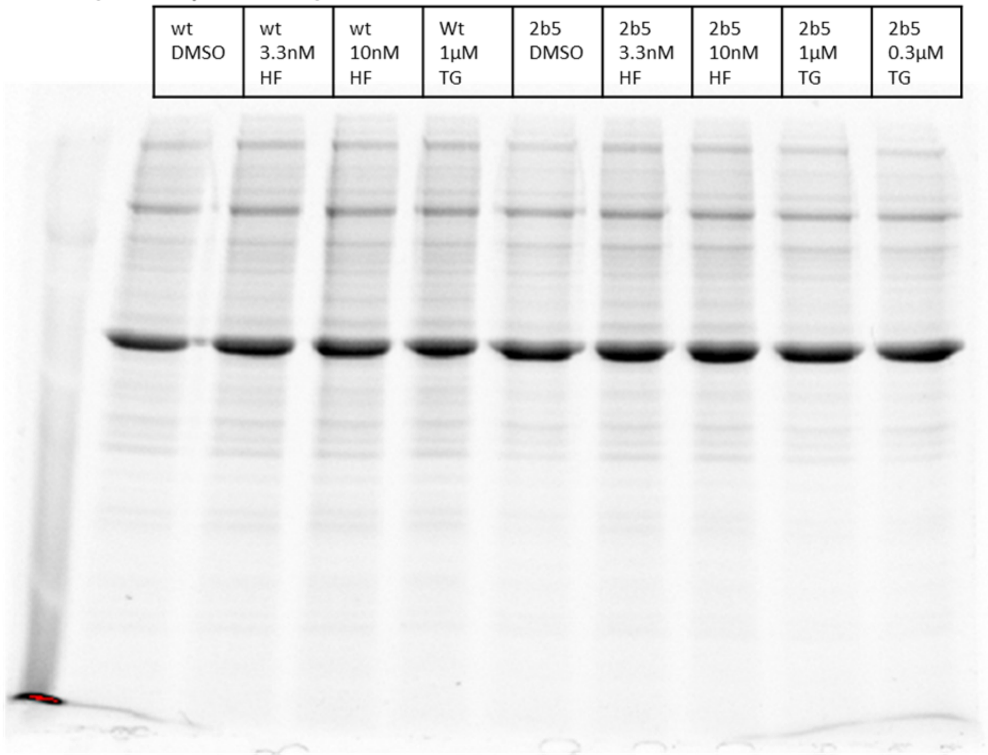

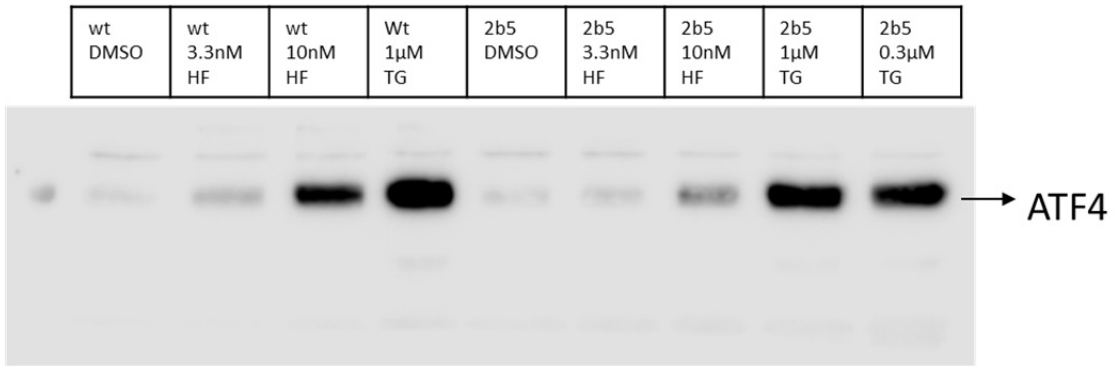

Overview of blots used for supplementary Fig. S1 (1/3)

TCE (total protein)

2b5 DMSO	2b5 3.3nM HF	2b5 10nM HF	2b5 1µM TG	wt DMSO	wt 3.3nM HF	wt 10nM HF	Wt 1µM TG	Wt 0.3µM TG

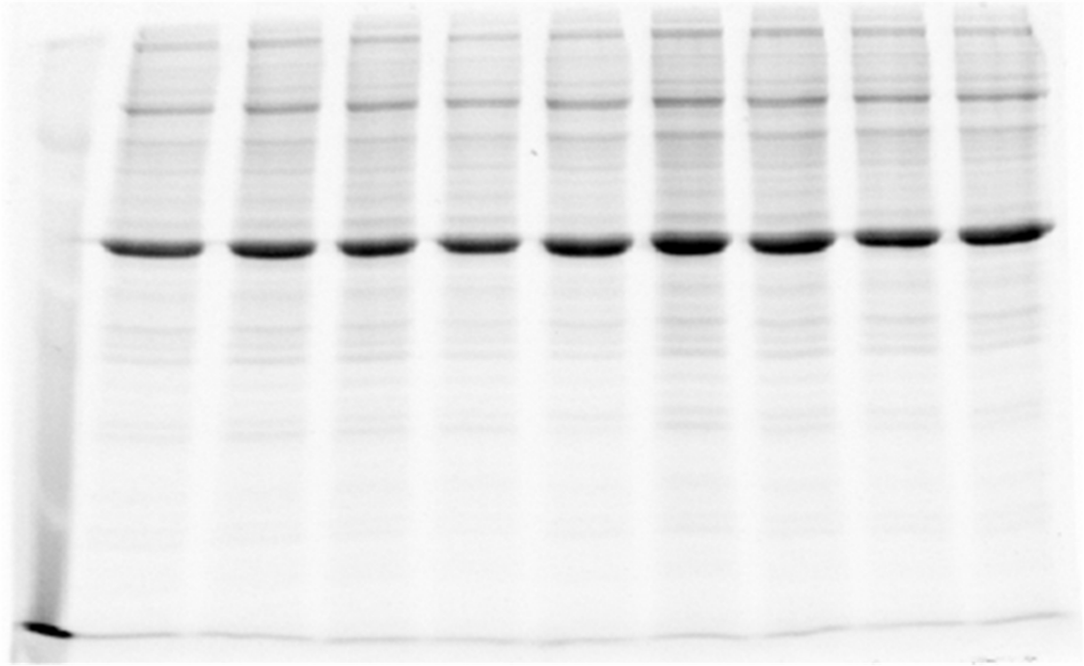

2b5 DMSO	2b5 3.3nM HF	2b5 10nM HF	2b5 1µM TG	wt DMSO	wt 3.3nM HF	wt 10nM HF	Wt 1µM TG	Wt 0.3µM TG

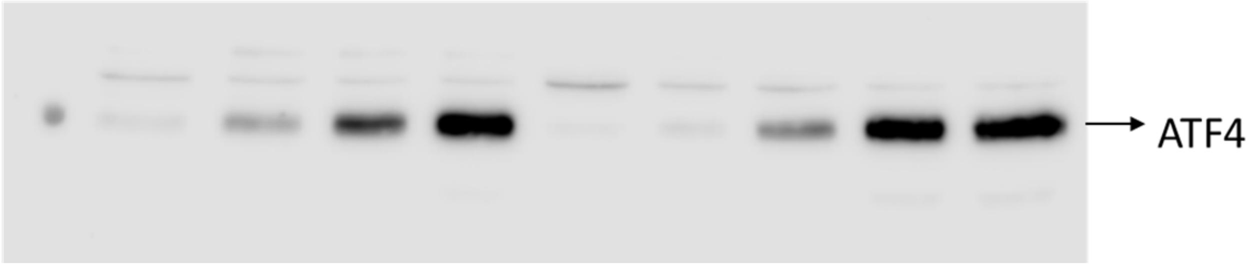

→ ATF4

Overview of blots used for supplementary Fig. S1 (2/3)

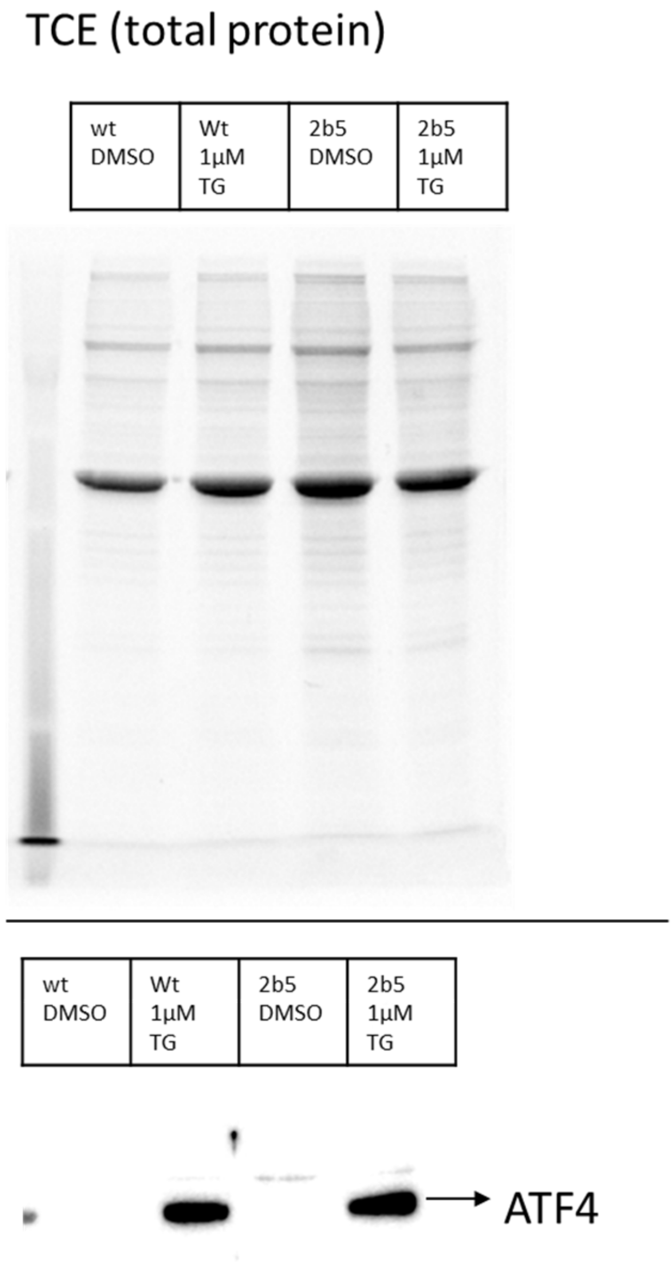

Overview of blots used for supplementary Fig. S1 (3/3). Top row contains the TCE stains (total protein) with the corresponding ATF4 immunoreactivity (detected with the SC-200 antibody) immediately below. Full blots are shown.
